# Identification of Pathogenic *Leptospira* Species in the Urogenital Tract of Water Buffaloes (*Bubalus bubalis*) From the Amazon River Delta Region, Brazil

**DOI:** 10.3389/fvets.2020.00269

**Published:** 2020-05-14

**Authors:** Israel Barbosa Guedes, Gisele Oliveira de Souza, Juliana Fernandes de Paula Castro, Antônio Francisco de Souza Filho, Matheus Burilli Cavalini, Sueli Akemi Taniwaki, Anderson Luiz Pinheiro Maia, Isaías Corrêa Pereira, Marcos Bryan Heinemann

**Affiliations:** ^1^Departamento de Medicina Veterinária Preventiva e Saúde Animal, Faculdade de Medicina Veterinária e Zootecnia, Universidade de São Paulo, São Paulo, Brazil; ^2^Médico Veterinário, Auditor Fiscal Agropecuário, Agência de Defesa e Inspeção Agropecuária Do Estado Do Amapá, Santana, Brazil; ^3^Secretaria de Estado da Saúde Do Amapá, Macapá, Brazil

**Keywords:** *Leptospira*, buffalo, PCR, DNA sequencing, Amazon

## Abstract

In the current context of deforestation and fire in the Amazon, buffaloes could be a cost-effective and sustainable alternative for cattle production in the region, as they can convert low-quality foods and be raised in floodplain areas. However, little is known about the reproductive diseases that affect these animals; thus, the purpose of this study was to perform the molecular characterization of *Leptospira* spp. in the urogenital tract of water buffaloes (*Bubalus bubalis*) raised in the Amazon River Delta region in Brazil. Samples were collected from 114 kidneys, 204 ovaries, and 160 uterine swabs of slaughtered buffaloes in the Macapá microregion of Amapá State (Brazil) and were subjected to PCR to detect bacterial DNA. Positive amplicons were sequenced to identify *Leptospira* species. Among the total samples, 11/473 were PCR positive (2.3%), including 10 kidney samples and one uterine swab sample. DNA sequencing identified two pathogenic species from the kidney samples: *L. interrogans*, accounting for 60.0% (6/10) of these samples, and *L. borgpetersenii*, accounting for 20.0% (2/10), while 20.0% (2/10) were identified only at the genus level. The bacterium in the uterine swab sample was identified as *L. interrogans* with genetic proximity to strains belonging to the serovar Hardjo. This is the first report of leptospires species identified in buffaloes from the Amazon River Delta region and revealed that these animals may be carriers of different pathogenic *Leptospira* species, similar to bovines, including showing genital colonization.

## Introduction

Leptospirosis is a bacterial disease that affects humans and several species of domestic and wild animals and is considered a zoonosis (1). The genus *Leptospira* can be considered to be dynamic and diversified in relation to the species it includes; with the advancement of molecular methodologies, it has been possible to define 64 species, which are now divided into two major clades, one of which contains pathogenic species, while the other contains saprophytes, leading to a new proposal for the systematic classification of the genus (2). Nevertheless, the serological classification of leptospires is still accepted and is the basis of serological assays such as the microscopic agglutination test (MAT), in which a limited number of serovars that represent prevalent serogroups for a specific region are used (1, 3).

Similar to the disease in cattle, buffalo leptospirosis is mainly characterized by reproductive disorders, especially abortions, in which *Leptospira* spp. have been detected (4, 5). In Brazil, there has been only one report of *Leptospira* isolation from the urine of a healthy buffalo from the southeastern region of the country, and the isolate was classified as *L. santarosai* serovar Guaricura (6). Due to the difficulties in isolating leptospires from biological samples, direct DNA sequencing from PCR products is used, which allows the identification of leptospires at the species level, providing a new epidemiological analysis of the disease (7–9).

In 2018, the Brazilian buffalo herd consisted of 1,390,066 buffaloes, and ~37% of this herd was concentrated in the Amazon River Delta region (considering the east coast of Amapá state and Marajó Island) (10). Buffaloes are part of the Amazonian culture since it is used as a work animal and it plays a role in tourism, so these animals are in close proximity to humans (11). This close contact may represent a public health problem since leptospirosis is a zoonosis, and it has been reported that buffaloes can be directly involved in the transmission of leptospirosis to humans (12). In this region, serological studies have revealed the presence of anti-*Leptospira* spp. antibodies in buffaloes, with a prevalence ranging from 34.37 to 80.0% and predominance of the Sejroe serogroup (13–15), similar to what is observed in cattle. Nevertheless, there have been few studies on leptospirosis in buffaloes; thus, the aim of this study was to detect and perform the molecular characterization of *Leptospira* spp. in the urogenital tract of buffaloes raised in the Amazon River Delta region, providing new knowledge about leptospirosis in these animals.

## Materials and Methods

This work was approved by the Ethics Committee on Animal Use of the School of Veterinary Medicine and Animal Science (Universidade de São Paulo)**—**CEUA/FMVZ n° 5613211118. For this study, 114 kidney fragments (~5 g), 204 ovaries and 160 uterine swabs were collected from buffaloes slaughtered in a slaughterhouse in the Macapá microregion of Amapá State, Brazil. Sampling was carried out by convenience, and for logistical reasons during slaughter, the samples were collected in groups on different days according to the type of tissue (kidneys**—**day 1, ovaries**—**day 2, uterine swabs**—**day 3); thus, each sample represented a single animal, totaling 478 animals.

**Figure 1:**
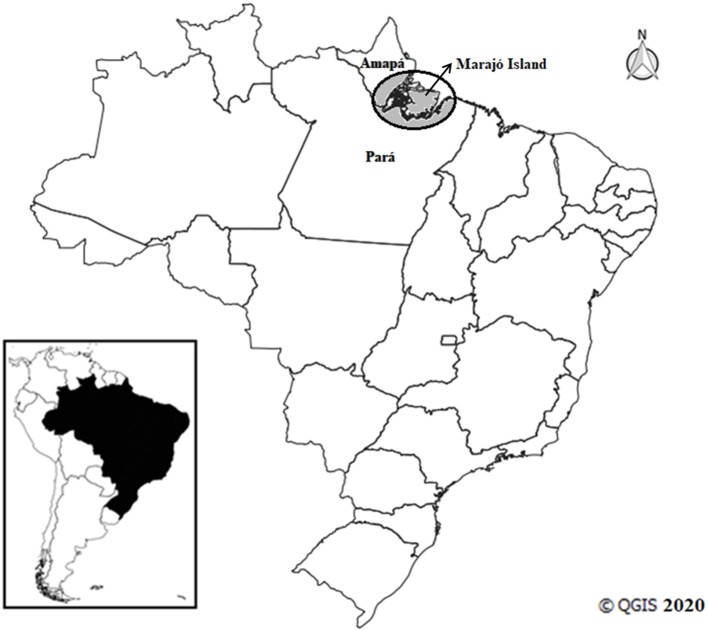
Amazon River Delta region in Brazil (circle).

The animals came from different farms located in the Amazon River Delta region in northern Brazil, which is characterized as a geographical area formed by hundreds of islands and islets between the states of Pará and Amapá, encompassing Marajó Island (Figure 1). The animals predominantly belonged to the River Buffalo group breeds (Murrah, Mediterranean, and Jafarabadi) and included males and females that were at least 12 months old, showed meat production capacity, had not been vaccinated against leptospirosis and had an unknown reproductive history.

During the slaughter of the animals, fragments of the kidneys, and ovaries were collected with the aid of sterile forceps and scissors and stored individually in sterile plastic bags for homogenization. At the slaughterhouse immediately after collection, the organs were macerated and diluted 1:10 in phosphate-buffered saline [PBS; 0.137 M NaCl; 0.0027 M KCl; 0.01 M Na_2_HPO_4_; and 0.0018 M KH_2_PO_4_ (pH 7.4)]. The uterine swab samples were obtained with the aid of a disposable cervical brush fixed to a swab, which was vigorously rubbed against the mucosa of the uterine horns and body, and were also diluted in phosphate-buffered saline. A 1 ml aliquot of the diluted samples was frozen and sent to the laboratory for tests.

The extraction and purification of DNA from the samples were performed using the PureLink® Genomic DNA Mini Kit (Invitrogen™) according to the manufacturer's protocol. PCR targeting the *Bubalus bubalis* cytochrome b (*cytb*) gene, as described by Bottero et al. (16), was used as an internal control to verify the validity of the extracted DNA. The detection of *Leptospira* spp. was carried out by PCR with the Lep1 and Lep2 primer pair, which amplifies a 330 bp region of the 16S rRNA gene (*rrs*) (17), using Go Taq™ Green Master Mix (Promega, Brazil). The positive samples were subjected to another round of PCR for typing using primer pairs that amplify a 549 bp region of the *secY* gene (18). *L. interrogans* serovar Hardjo-prajitno and ultrapure water were used as positive and negative controls, respectively. A negative control sample was inserted between every five test samples to assess the presence of contamination in the extracted DNA.

**Figure 2:**
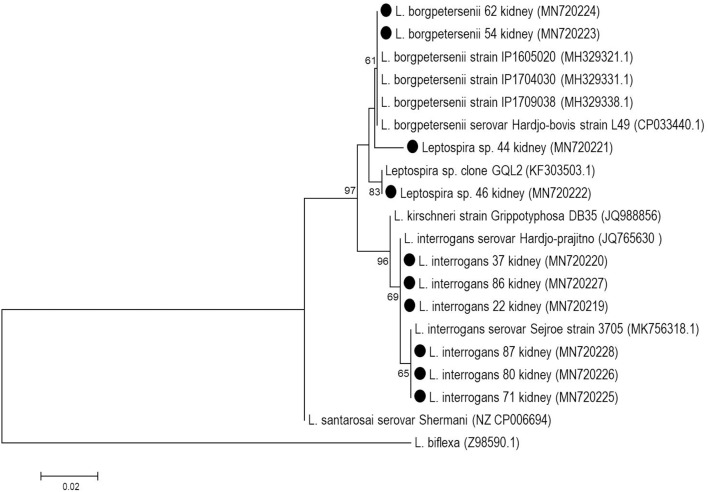
Phylogenetic reconstruction based on partial *rrs* gene of *Leptospira* spp. from kidney samples of buffaloes (black circles). The tree was elaborated with Neighbor-Joining method, Tamura-3 parameter model with bootstrap test of 1,000 replicates.

**Figure 3:**
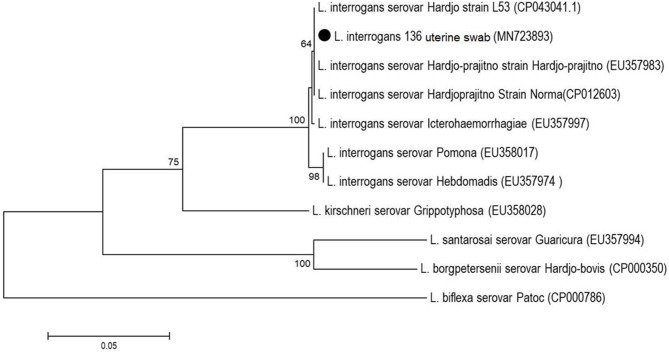
Phylogenetic analysis based on partial *secY* gene of *Leptospira* spp. from the uterine swab sample (black circle). The tree was elaborated with Neighbor-Joining method and Tamura-3 parameter model with bootstrap test of 1,000 replicates.

Positive amplicons were sequenced by the Sanger method with BigDye Terminator v.3.1 chemistry (Applied Biosystems) and an ABI-3500 (Applied Biosystems) automatic sequencer according to the manufacturer's instructions. The sequences were assembled with the BioEdit Sequence Alignment Editor (19). The phylogenetic trees were built using homologous sequences retrieved from the GenBank database (accession numbers in Figures 2, 3) with the neighbor-joining method, the Tamura-3-parameter model and 1,000 bootstrap replicates in MEGA 7 software (20).

## Results

Among the total samples (478) subjected to the internal control PCR assay, only five ovarian samples were not amplified and were disregarded, resulting in a total of 473 samples that were analyzed. In the *rrs* PCR analysis, 11/473 samples were positive (2.3%), 10 of which were kidney samples, and one was a uterine swab. All ovarian samples were negative. None of these positive kidney samples showed amplification of the *secY* gene. However, the *rrs*-positive amplicons were sequenced (GenBank accession numbers from MN720219 to MN720228), and it was possible to identify two pathogenic species: *L. interrogans*, accounting for 60.0% (6/10) of these samples, and *L. borgpetersenii*, accounting for 20.0% (2/10), while 20.0% (2/10) of the samples were identified only to the genus level and also corresponded to pathogenic species (Figure 2).

The positive uterine swab sample was identified as *L. interrogans* by the partial sequencing of the *secY* gene (GenBank accession number: MN723893) and showed genetic proximity to strains belonging to the serovar Hardjo (Figure 3). As each sample represented a single animal, it was not possible to correlate the samples with each other.

## Discussion

Although buffaloes are considered rustic, as reflected in their natural resistance to disease in the environment, these animals can be affected by infectious agents, including those related to reproductive disorders (21). In this work, the identification of bacterial DNA in animals showed that buffaloes may be as sensitive to *Leptospira* spp. as cattle, including showing renal colonization and, consequently, the elimination of bacteria through the urine, which becomes a source of environmental contamination (22).

In the Amazon, climatic conditions may favor the occurrence of leptospirosis in cattle and buffalo, since the risk of animals contracting the disease is higher in regions affected by seasonal flooding (23). In bovines, the control of leptospirosis is difficult because different strains of leptospires may be adaptable to cattle (24) and the disease is not yet well-understood in buffalo, as few studies have provided information about the species of *Leptospira* circulating in these animals.

The direct sequencing of PCR products from kidney samples showed that the pathogenic species *L. interrogans* was most prevalent (6/10) in these samples, whereas the *L. borgpetersenii* is usually reported to be the most prevalent species in cattle (7, 9). In other animal species such as ewes and horses, *L. interrogans* is most commonly found in the genital tract (25, 26); in association with such infections, recurrent uveitis, a classic leptospirosis injury in horses, seems to be directly related to strains of *L. interrogans* (27). Two sequences (MN720221 and MN720222) formed two independent clades and could not be identified at the species level, but is suggested that this two samples are *L. borgpetersenii*-like species, as these two samples showed >98.9% identity with *L. borgpetersenii*; in particular the MN720222 sample was homologous to *L. borgpetersenii* isolated from a human patient in Guayaquil, Ecuador (28).

A difficulty found in the present study was that the kidney samples positive for the *rrs* gene could not be amplified by PCR targeting the *secY* gene. This situation has also been observed in other studies, and the discrepancy between PCR assays could arise due to the amount of original bacterial DNA in the material as well as the quality of the sample tested (7, 9). In addition, not all PCR products can always be sequenced (29) or generate interpretable data (30).

Despite the limitations of the use of the *rrs* gene to separate *Leptospira* species (2), this target and *secY* gene, which are used for the molecular characterization of leptospires, have many characterized samples deposited on GenBank which facilitates sample typing (8, 31–33). Increasingly, different target genes have been described and proposed to assist in the molecular characterization of leptospires (*lipL32, flaB, gyrB, rpoB*, among others) which had success both in characterizing isolates and directly from PCR products, although this last option is less sensitive. These targets can generate “barcodes” which have variable discriminatory power, and can be combined to increase the differentiation of *Leptospira* species (34).

An interesting result was that *L. santarosai* was not detected in the buffalo samples, differing from what is reported for cattle in other regions of the country, such as the southeast, where this species is one of the most prevalent (8, 24, 35). The dimensions and heterogeneity of the with climatic conditions in the Amazon make this region unique and different from other parts of the country, which is reflected in the etiological agent-host relationships and may account for the situation found in cattle from the Amazon region, where *L. santarosai* may not be involved in leptospirosis in these animals (9, 36). Regarding buffalo, more work is needed to evaluate the participation of *L. santarosai* and others species in buffalo leptospirosis.

No ovarian specimens were PCR positive, suggesting that this organ is not be a preferred site for the colonization of leptospires in buffalos, although there has been one report of leptospiral DNA detected in the ovaries of slaughtered sheep (37). The isolation of *L. interrogans* from ovaries has been reported, but only in experimentally infected hamsters in the acute phase of the disease (38). The potential adaptability of leptospires to their hosts, especially in cattle, leads to a chronic character of these infections, where extrarenal colonization by leptospires has been described focusing on the structures of the female reproductive system, such as the vagina, uteri, and oviducts (39–41).

The positive uterine swab sample (MN723893), belonging to *L. interrogans*, showed homology to sequences characterized as belonging to serovar Hardjo strains, including an autochthonous sample (CP043041.1) isolated from the urine of dairy cattle in Paraná, in the southern region of Brazil (42). This finding may suggest that sexual transmission may be occurring among these animals. The presence of *Leptospira* in the reproductive tract of animals (25, 40, 41) demonstrates the tissue tropism of the bacterium, causing reproductive disorders, especially the strains of the Sejroe serogroup, which are considered to be adapted to cattle, promoting chronic infection of the genital tract and subtly compromising the reproductive performance of these animals for long periods. This condition has recently been described as a syndrome, referred to as bovine genital leptospirosis (BGL) (43).

## Conclusion

This is the first report of leptospires species identified in buffaloes from the Brazilian Amazon and revealed that these animals may be carriers of different pathogenic *Leptospira* species, similar to bovines. Genital colonization was found, showing that the reproductive system of the buffaloes may be affected.

## Data Availability Statement

The datasets generated for this study can be found in the Genbank NCBI. Accession numbers from MN720219 to MN720228 and Genbank accession number: MN723893.

## Ethics Statement

The animal study was reviewed and approved by Ethics Committee on Animal Use of the School of Veterinary Medicine and Animal Science (University of São Paulo).

## Author Contributions

IG, AM, and IP carried out the collection of samples at the slaughterhouse. IG, GO, JF, AF, and MC performed all the laboratory tests. IG and ST performed the interpretation of DNA sequencing results. IG wrote the manuscript and ST did the translation. MH accurately reviewed the manuscript. All authors have read and approved the final version of the manuscript.

## Conflict of Interest

The authors declare that the research was conducted in the absence of any commercial or financial relationships that could be construed as a potential conflict of interest.
